# Piezo2 is not an indispensable mechanosensor in murine cardiomyocytes

**DOI:** 10.1038/s41598-022-12085-9

**Published:** 2022-05-17

**Authors:** Benjamin Kloth, Giulia Mearini, Florian Weinberger, Justus Stenzig, Birgit Geertz, Jutta Starbatty, Diana Lindner, Udo Schumacher, Hermann Reichenspurner, Thomas Eschenhagen, Marc N. Hirt

**Affiliations:** 1 Institute of Experimental Pharmacology and Toxicology, University Medical Center Hamburg-Eppendorf, Martinistraße 52, 20246 Hamburg, Germany; 2 Department of Cardiac Surgery, University Heart & Vascular Center, University Medical Center Hamburg-Eppendorf, Hamburg, Germany; 3 DZHK (German Centre for Cardiovascular Research), Partner Site Hamburg/Kiel/Lübeck, Hamburg, Germany; 4 Department of Cardiology, University Heart & Vascular Center, University Medical Center Hamburg-Eppendorf, Hamburg, Germany; 5 Institute of Anatomy and Experimental Morphology, University Medical Center Hamburg-Eppendorf, Hamburg, Germany

**Keywords:** Cardiac hypertrophy, Cardiovascular genetics

## Abstract

A short-term increase in ventricular filling leads to an immediate (Frank-Starling mechanism) and a slower (Anrep effect) rise in cardiac contractility, while long-term increased cardiac load (e.g., in arterial hypertension) decreases contractility. Whether these answers to mechanical tension are mediated by specific sensors in cardiomyocytes remains elusive. In this study, the piezo2 protein was evaluated as a potential mechanosensor. Piezo2 was found to be upregulated in various rat and mouse cardiac tissues upon mechanical or pharmacological stress. To investigate its function, C57BL/6J mice with homozygous cardiomyocyte-specific piezo2 knockout [Piezo2-KO] were created. To this end, α-MHC-Cre mice were crossed with homozygous “floxed” piezo2 mice. α-MHC-Cre mice crossed with wildtype mice served as controls [WT-Cre^+^]. In cardiomyocytes of Piezo2-KO mice, piezo2 mRNA was reduced by > 90% and piezo2 protein was not detectable. Piezo2-KO mice displayed no morphological abnormalities or altered cardiac function under nonstressed conditions. In a subsequent step, hearts of Piezo2-KO or WT-Cre^+^-mice were stressed by either three weeks of increased afterload (angiotensin II, 2.5 mg/kg/day) or one week of hypercontractility (isoprenaline, 30 mg/kg/day). As expected, angiotensin II treatment in WT-Cre^+^-mice resulted in higher heart and lung weight (per body weight, + 38%, + 42%), lower ejection fraction and cardiac output (− 30%, − 39%) and higher left ventricular anterior and posterior wall thickness (+ 34%, + 37%), while isoprenaline led to higher heart weight (per body weight, + 25%) and higher heart rate and cardiac output (+ 24%, + 54%). The Piezo2-KO mice reacted similarly with the exception that the angiotensin II-induced increases in wall thickness were blunted and the isoprenaline-induced increase in cardiac output was slightly less pronounced. As cardiac function was neither severely affected under basal nor under stressed conditions in Piezo2-KO mice, we conclude that piezo2 is not an indispensable mechanosensor in cardiomyocytes.

## Introduction

The heart adapts its performance to meet the body’s demands. As some of this regulation is fully autonomous (even in denervated or explanted hearts), the heart itself must have the ability to perceive mechanical forces. An immediate length-dependent increase of contractility upon increased ventricular filling is named after its discoverers: the Frank-Starling mechanism. The fact that a variety of theories exist that try to explain this phenomenon indicates that it is not yet fully understood. However, an increase in myofilament Ca^2+^ sensitivity and the role of the largest known protein titin as a pivotal stress sensor appear to be essential^1,2^. Additionally, a slower force increase over 10–15 min is known as the Anrep effect. A series of events starting with the release of angiotensin II by stretch, followed by endothelin release, mineralocorticoid receptor activation, epidermal growth factor receptor transactivation, mitochondrial ROS release, activation of the Na^+^/H^+^-exchanger (NHE1) and ultimately an increase in Ca^2+^-transient amplitude through the Na^+^/Ca^2+^-exchanger has been postulated as a molecular explanation^3^. However, the initial stretch-mediated Ang II release largely remains a black box. Early reports described the release of preformed Ang II in cardiomyocytes upon stretch^4,5^, but others favour a direct stretch-mediated activation of the Ang II type 1 receptor without physical release of AT II^6^. The latter is an example of the current paradigm of how cardiomyocytes perceive mechanical forces, namely, by a secondary function of G-protein coupled receptors but also of structural proteins or complexes (e.g., microtubules^7^, integrins^8^, desmosomes and fasciae adherentes^9^), ion channels^10^, sarcomeric proteins (e.g., titin^1,2^) or other structures (e.g., caveolae^11^).

In addition to the Frank-Starling and Anrep mechanisms as short-term adaptations to varying cardiac load, long-term adaptations occur in response to physiological challenges such as endurance training or pregnancy, but also to pathological stress factors (e.g., arterial hypertension or aortic stenosis). The latter primarily affects the heart due to an increase in afterload. Whether this enhanced cardiac wall stress is perceived by additional sensory capabilities of existing structures in cardiomyocytes remains uncertain.

Here, we hypothesized that cardiomyocytes have specialized mechanosensors and focused on piezo proteins (in particular piezo2). Both members of this family (piezo1 and 2) had been identified a decade ago in a screening for genuine mechanosensors^12^, and mutations in both genes have been associated with cardiovascular disease, including heart failure^13,14^.

## Methods

### Generation, culture and analysis of rat engineered heart tissue (rEHT)

The workflow to generate rat EHT has been described in detail previously^15,16^. Briefly, EHT casting molds in a 24-well format were prepared by putting polytetrafluoroethylene spacers into liquid, 60 °C warm 2%-agarose. After solidification, spacers were removed and a silicone rack with two hollow silicone posts protruding into each mold was positioned on top of the culture dish. Hearts of neonatal rats (postnatal day 0 to 3) were excised, and ventricular rat heart cells (all cell types, but moderately enriched for cardiomyocytes)^17^ were isolated by trypsin/DNAse digestion. A total of 500,000 cells per EHT were mixed with fibrinogen and thrombin at a final volume of 100 µL. This mixture was quickly pipetted into the casting molds. After 90 min, the fibrin block containing the heart cells could be removed from the molds with the help of the two silicone posts, in between which the fibrin strip was spanned. These EHTs were transferred to 24-well culture dishes filled with medium consisting of low glucose (1 g/L) DMEM (Biochrom F0415), 10% inactivated horse serum (Gibco 26050), penicillin/streptomycin (each 100 U/mL, Gibco 15140), insulin (10 µg/mL, Sigma-Aldrich I9278) and aprotinin (33 µg/mL, Sigma-Aldrich A1153, to prevent rapid fibrin degradation). During a culture period of two weeks, EHTs started to beat coherently. The medium was changed three times per week. In the third week of culture, half of the EHTs were mechanically challenged. This procedure is called afterload enhancement (AE) procedure and has been described in detail elsewhere^16^. In brief, the silicone posts representing both anchoring points and elastic resistance for the attached EHT were stiffened by metal braces. This procedure increased the afterload of EHTs by a factor of 12, which leads, e.g., to cardiomyocyte hypertrophy and contractile dysfunction.

All EHT-related animal work was reviewed and approved by the regional ethics review board of the Medical Council of Hamburg, Germany (approval number ORG516) and was conducted in accordance with the Guide for the Care and Use of Laboratory Animals as adopted by the United States National Institutes of Health (8th edition, revised 2011) and with the ARRIVE (Animal Research: Reporting of in Vivo Experiments) guidelines.

### TAC-surgery in mice and rats

At the age of 8 to 9 weeks, male C57BL/6J mice underwent transverse aortic constriction (TAC) or sham operation. This was performed by constricting the aortic arch between the brachiocephalic trunk and the left carotid artery. Therefore, mice were anesthetized and mechanically ventilated. Hereafter, the chest was entered by an upper partial sternotomy, and a suture was tied around the aortic arch against a 27-gauge cannula^18^. After removing the cannula, the inner aortic diameter was constricted by approximately 70%^19^. The follow-up period was either 2 or 6 weeks.

Mice were sacrificed, and after cardiac explantation, the atria and the right ventricle were removed. The remaining left ventricle was immediately snap-frozen in liquid nitrogen and stored at − 80 °C for further analysis.

TAC surgery in male Wistar rats was performed at an age of 3 weeks. Again, the aortic arch was constricted mechanically between the brachiocephalic trunk and the left carotid artery. To this end, rats underwent isoflurane inhalation anesthesia, mechanical ventilation and hemisternotomy to expose the aortic arch. For rat TAC, an incompletely closed vascular clip was placed around the aorta as described by Zaha et al.^20^ The clip applicator device (Horizon Metal Ligation System, Teleflex, Morrisville, NC, USA) was fitted with a custom-made stopper device to ensure a consistent remaining diameter. The inner diameter of the clip was set to 0.45 mm. After surgery, rats were treated twice daily with buprenorphine and carprofen and sacrificed after 4 weeks. Hearts were immediately excised, relaxed in 20 mM KCl solution and shock frozen in liquid nitrogen.

All investigations were approved by the regulatory authorities of Hamburg, Germany (G13/067, G13/115, #08/14) and were conducted in accordance with the Guide for the Care and Use of Laboratory Animals as adopted by the United States National Institutes of Health (8th edition, revised 2011) and with the ARRIVE (Animal Research: Reporting of In Vivo Experiments) guidelines.

### Creation of cardiomyocyte specific piezo2 knock-out-mice

Floxed piezo2-mice (piezo2fl^/fl^) were kindly provided by the Patapoutian lab (Scripps Research, La Jolla, CA, USA). Piezo2^fl/fl^ mice contain two loxP sites flanking exons 43 through 45. This region close to the C-terminus is highly conserved across different species, and Cre excision of exons 43–45 causes a frameshift mutation in piezo2, introducing an early stop codon^21^. The mice were created on a C57BL/6N background and had normal piezo2 expression and no pathological phenotype. They were transferred to a C57BL/6J background by 5 consecutive crossbreeds and finally crossed with α-MHC-Cre mice, which were also kept on a C57BL/6J background. In α-MHC-Cre mice, the expression of Cre recombinase from bacteriophage P1 is driven by the cardiomyocyte-specific α-MHC promoter, which is briefly activated during cardiac development, always activated in the atria and postnatally rapidly and permanently activated in the ventricles^22,23^. By crossbreeding, we created four different groups of mice: (1) piezo2^fl/fl^ X α-MHC-Cre^+^ [Short name: Piezo2-KO], (2) piezo2^wt/wt^ X α-MHC-Cre^+^ [Short name: WT-Cre^+^], (3) piezo2^wt/fl^ X α-MHC-Cre^+^ [Short name: Het Piezo2-KO] and (4) piezo2^fl/fl^ X α-MHC-Cre^−^^22,24^.

### Implantation of osmotic minipumps

Osmotic minipumps were implanted into C57BL/6J WT mice or WT-Cre^+^ and Piezo2-KO mice at the age of 10 to 12 weeks. To increase cardiac afterload of mice, angiotensin II at 2.5 mg/kg/per day was loaded into model 2004 pumps (ALZET), which stayed in place for three weeks^25^. To decrease afterload and induce tachycardia, isoprenaline at 30 mg/kg/per day was loaded into model 1007D pumps (ALZET), which stayed in place for one week only. Minipumps of the respective subtypes loaded with isotonic NaCl were implanted into control animals.

For the implantation mice were anesthetized. A subcutaneous pocket was created by a small mid-scapular incision, and the minipump was inserted, with the delivery portal first. The wound was closed with clips. After the experimental period, the mice were sacrificed, the hearts were explanted, the atria were removed, and the remaining ventricles were immediately snap-frozen in liquid nitrogen and stored at − 80 °C for further analyses.

All investigations were approved by the local regulatory authorities of Hamburg, Germany (G13/115, # 93/15) and were conducted in accordance with the Guide for the Care and Use of Laboratory Animals as adopted by the United States National Institutes of Health (8th edition, revised 2011) and with the ARRIVE (Animal Research: Reporting of in Vivo Experiments) guidelines.

### Echocardiography

Transthoracic echocardiography was performed prior to and at the end of the follow-up period. Mice were anesthetized in an induction chamber. The fur was removed from the neckline to the mid chest using a shaver and hair removal cream. Anaesthetized mice were placed in a supine position atop a heating pad with the snout in a nose cone connected to the anesthesia system. Transthoracic echocardiography was performed using the Vevo 3100 System (VisualSonics). Images were obtained in the parasternal short- and long-axis view. The dimensions of the left ventricle (thickness of the anterior and posterior walls, as well as the left ventricular diameter) were measured in the short axis view during both diastole and systole.

### Immunohistochemistry (IHC) and immunofluorescence (IF)

Rat EHTs, rat hearts or mouse hearts were fixed for at least 24 h in a phosphate-buffered solution containing 4% formaldehyde stabilized with methanol. After embedding in paraffin, 4–5 µm sections were cut (for EHTs longitudinally in the median plane). Staining conditions for piezo2 were optimized on rat heart tissue samples and worked for mouse and rat tissues. After antigen retrieval (30 min [IHC] or 15 min [IF] in citrate buffer, pH 6.0), rabbit anti-piezo2 polyclonal antibody (Sigma Life Science, HPA031974) at a dilution of 1:1000 [IHC] or 1:100 [IF], mouse anti-troponin I monoclonal antibody (Merck, MAB 1692) at 1:1000 [IF], mouse anti-α-smooth muscle actin monoclonal antibody (R&D, MAB1420) at 1:200 [IF] or IgG isotype (as a control) was employed. Nuclei were stained with hematoxylin [IHC] or DAPI 1:1000 [IF] and cell membranes with wheat germ agglutinin, Alexa Fluor 633 Conjugate (Invitrogen, W21404) 1:400 [IF].

Antibodies were either visualized with the multimer technology-based UltraView Universal Alkaline Phosphatase Red Detection Kit (Roche, 760–501 for IHC) or with goat anti-rabbit Alexa Fluor 488 (Invitrogen, 11034)/goat anti-mouse Alexa Fluor 546 (Invitrogen, 11003) for IF at a dilution of 1:100.

### Statistics

Results are presented as mean ± SEM. All graphs and statistical tests were created or performed in GraphPad Prism version 8.43. Student’s unpaired t-test was applied to compare two groups. For more than two groups, appropriate ANOVAs and Sidak’s corrections for multiple comparisons were performed. Adjusted p < 0.05 or less was considered statistically significant. P-values are displayed graphically as follows: *p < 0.05, **p < 0.01, ***p < 0.001, ns = not significant.

## Results

### Mechanical stress leads to upregulation of piezo2 in cardiac tissue

**Figure 1:**
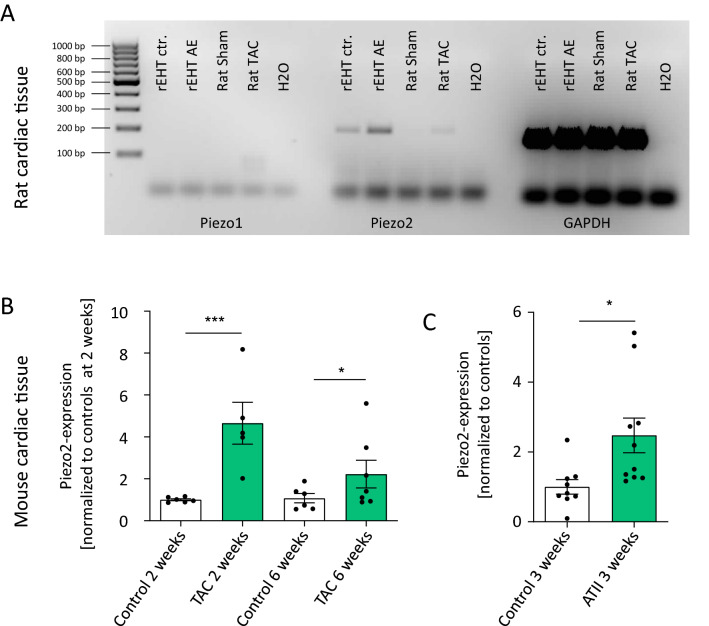
Upregulation of piezo2 in different cardiac stress models. (**A**) Semiquantitative PCRs for piezo1, piezo2 and GAPDH of control and afterload-enhanced (AE) rat EHTs as well as rat left ventricles after sham operation or TAC (transverse aortic constriction) surgery. Each lane represents the PCR-product from one sample (rat or EHT). The expected or detected PCR product sizes were 167 bp (piezo1), 200 bp (piezo2) and 155 bp (GAPDH). (**B**) Quantitative PCR for piezo2 expression in the left ventricles of control mice or after 2 or 6 weeks of TAC (n = 5–7). (**C**) Quantitative PCR for piezo2 expression in the left ventricles of control mice or after 3 weeks of continuous angiotensin II (ATII) application (n = 9–10). In (**B**, **C**) each dot represents the cardiac piezo2-expression of one mouse, averaged from two technical replicates. For better visualization the mean of control 2 weeks (**B**) or control 3 weeks (**C**) was set to 1.

In several models, we investigated whether mechanical or pharmacological stress led to an upregulation of piezo proteins in cardiac tissue. One model was developed and published by our group and is based on a week-long afterload enhancement (AE) of spontaneously beating rat engineered heart tissue (EHT), which leads to typical characteristics of pathological cardiac hypertrophy^16^. In a review of our published microarray data^17^, only piezo2 was sufficiently highly expressed to be analyzed and was upregulated by afterload increase (fold change [AE vs. control EHT] = 1.65, p = 0.045, n = 5 per group). These findings were confirmed with piezo1- and piezo2-specific primers by semiquantitative PCR (Fig. 1A). Left ventricular tissues from rats 4 weeks after transverse aortic constriction (TAC) also showed higher piezo2 expression after cardiac stress than sham-operated rats (Fig. 1A). Accordingly, we focused on piezo2 and additionally investigated its expression in murine tissue. Here, again, TAC animals exhibited higher piezo2 expression, which was more pronounced 2 weeks than 6 weeks after TAC (Fig. 1B). Last, the implantation of angiotensin II-releasing osmotic minipumps into mice, which led to a pharmacologically mediated increase in cardiac afterload, led to a comparable increase in piezo2 expression (Fig. 1C).

### Piezo2 is present at low mRNA and moderate protein levels in cardiomyocytes

**Figure 2:**
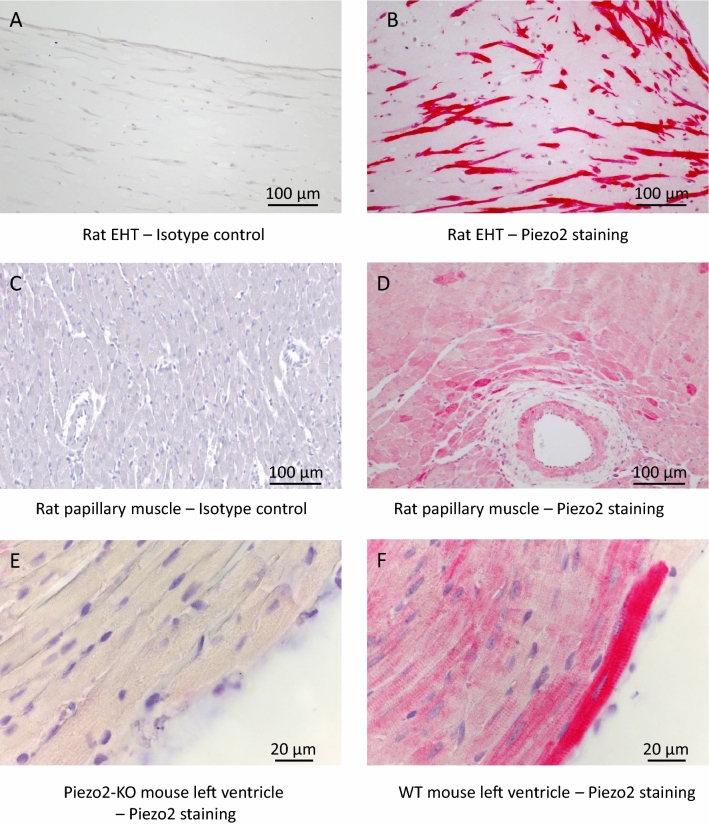
Immunohistochemical visualization of piezo2 in cardiac tissues. (**A**) Isotype control and (**B**) piezo2-staining of rat EHT. (**C**) Isotype control and (**D**) piezo2 staining of a rat papillary muscle. Note that endothelial cells and smooth muscle cells were also positive for piezo2. (**E**) Ventricular tissue of a Piezo2-KO and (**F**) a wildtype mouse stained for piezo2. Nuclei were counterstained with hematoxylin (blue) in all panels.

The above-described upregulation of piezo2 upon stress was observed in cardiac tissue comprising all cardiac cell types. Before investigating the potential role of piezo2 in cardiomyocytes, we analyzed its mRNA or protein abundance in this cell type. Published single-cell sequencing data from murine^26^ or human^27^ hearts display low but confidently detectable averaged piezo2 mRNA concentrations in cardiomyocytes (≈ 5% of cardiomyocytes show comparatively high values in the GTEx Project of the NIH) as well as in other cardiac cell types. Immunohistochemical staining for piezo2 revealed strong signals in cardiomyocytes of rat EHT (Fig. 2A,B). In native rat (Fig. 2C,D) and mouse (Fig. 2E,F) cardiac tissue, we observed a patchy cardiomyocyte staining pattern. The highest piezo2-abundance was observed around larger vessels and particularly in areas close to the tissue surface (Fig. S1). Besides cardiomyocytes (Fig. S2A) other cardiac cell types, such as endothelial cells and smooth muscle cells (Fig. S2B), were positive for piezo2. The staining patterns in mouse and rat cardiac tissues resembled those of human hearts published in “The Human Protein Atlas”^28^.

### Cardiomyocyte-specific piezo2 knockout mice are viable and do not have an overt phenotype

**Figure 3:**
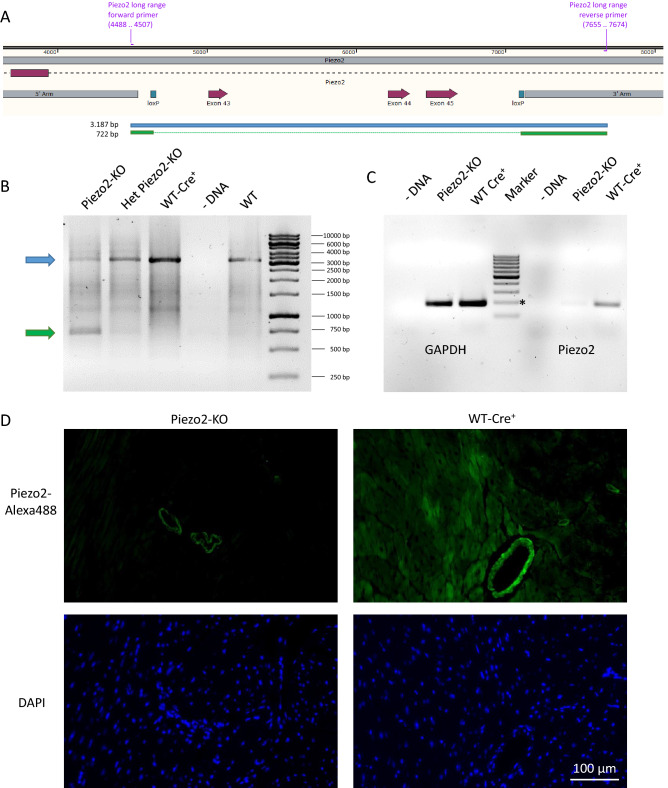
Cardiomyocyte specific knockout of piezo2. (**A**) Map of the floxed locus of piezo2^fl/fl^-mice. The excision of the interloxP region containing exons 43–45 leads to a frameshift mutation and the creation of a stop codon in the piezo2 gene. Blue bar: 3187 bp PCR product without recombination; green bar: 722 bp PCR product after successful recombination. (**B**) PCR of DNA from unpurified left ventricular tissue (i.e., containing all cardiac cell types) with long-range primers (delineated in **A**) followed by agarose gel separation. The blue and green arrows correspond to the sizes of the PCR products in (**A**). One representative lane for each mouse line is shown, overall n = 11 mice were analyzed. (**C**) Gel separation of the RT-PCR products from GAPDH or piezo2 mRNA obtained from purified cardiomyocytes. For each lane the PCR-products from the cardiomyocytes of one mouse heart were applied, for the quantification n = 3 per group were used. The molecular marker ranges from 100 to 1,000 bp in steps of 100. The GAPDH PCR product size was 185 bp, and the piezo2 PCR product size was 182 bp (*200 bp). (**D**) Immunofluorescence staining of Piezo2-KO and WT-Cre^+^-mice (green = piezo2, blue = nuclei).

To investigate the role of piezo2 in cardiomyocytes, we engineered cardiomyocyte-specific piezo2 knockout mice by crossing floxed piezo2 mice (Fig. 3A) with α-MHC-Cre mice. Overall, four different mouse groups were created: (1) piezo2^fl/fl^ X α-MHC-Cre^+^ [short name: Piezo2-KO], (2) piezo2^wt/wt^ X α-MHC-Cre^+^ [short name: WT-Cre^+^], (3) piezo2^wt/fl^ X α-MHC-Cre^+^ [short name: Het Piezo2-KO] and (4) piezo2^fl/fl^ X α-MHC-Cre^-^. The creation of WT-Cre^+^-mice was driven by evidence for chronic cardiac toxicity of simple Cre expression in α-MHC-Cre-mice^22,24^. To avoid attributing the supposed Cre toxicity to the piezo2-knockout, we used WT-Cre^+^-mice as default control.

The removal of the interloxP region containing exons 43–45 of piezo2 was verified by long-range PCR, which produced a short 722 bp PCR product when the region was successfully deleted or a long 3187 bp product when no deletion occurred. Unpurified DNA from left ventricular tissue (i.e., containing other cell types beside cardiomyocytes) was used for PCR. As expected, only the long fragment was detected in WT-Cre^+^. While there was only a small amount of the floxed fragment in Het Piezo2-KO, the floxed fragment constituted the dominant form in Piezo2-KO (Fig. 3B). The expected sequences of both PCR products were verified by Sanger sequencing.

In the next step, we investigated whether the piezo2 knockout in cardiomyocytes led to the eradication of piezo2 mRNA. To this end, cardiomyocytes from WT-Cre^+^ and Piezo2-KO mice were isolated in a Langendorff heart perfusion setup and purified by albumin gradient centrifugation. Only 9.4% of piezo2 mRNA (compared to WT-Cre^+^-mice) could be detected in Piezo2-KO mice (Fig. 3C). Piezo2 immunofluorescence staining of heart sections from Piezo2-KO (Fig. 3D) mice revealed no cardiomyocyte signal, while smooth muscle cells were clearly positive. The WT-Cre^+^ controls displayed both a patchy cardiomyocyte pattern (similar to Fig. 2D + F) and positive staining for smooth muscle cells.

Neither the heterozygous (Het Piezo2-KO) nor the homozygous (Piezo2-KO) piezo2-KO mice displayed any morphological or behavioral abnormalities under non-stressed conditions. A limited number of animals were also investigated by exploratory echocardiography, but no functional or morphological difference from WT mice could be observed (Fig. S3).

### Piezo2 knockout mice do not differ significantly from wild-type (Cre^+^) mice in the response to chronic angiotensin II or isoprenaline stimulation

Subsequently, we exposed WT-Cre^+^ and Piezo2-KO mice to two different pharmacological cardiac stress regimens. In the first arm of the study, angiotensin II was employed to evoke vasoconstriction and thus increase cardiac afterload for three weeks. In the second arm, isoprenaline served as hyperdynamic stress, which should lead to tachycardia (via β_1_-receptor stimulation) and vasodilatation and thus decreased afterload (via β_2_-receptor stimulation) for one week. Both drugs were administered via osmotic minipumps, and NaCl-loaded animals served as controls. To save animals, the Piezo2-KO + NaCl group was shared between both arms. All quantitative analyses were performed on the last day of drug treatment, i.e., at day 21 for angiotensin II and day 7 for isoprenaline. Additionally, the pre-treatment analyses as well as an extended set of post-treatment parameters are available in the Supplementary Material.

**Figure 4:**
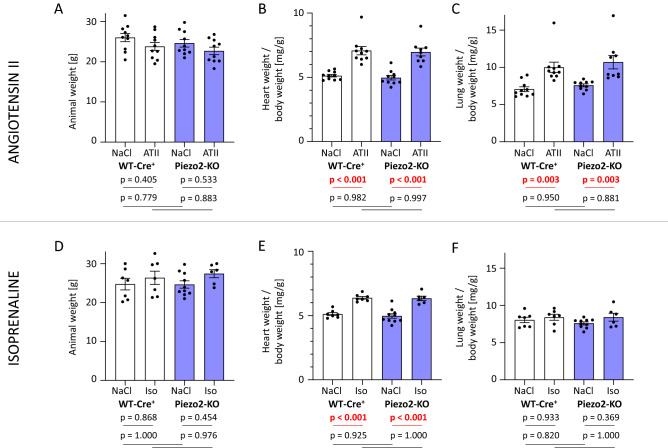
Animal or organ weights of WT-Cre^+^ and Piezo2-KO mice after angiotensin II or isoprenaline treatment. (**A**) Animals were weighed, and (**B**) heart weight/body weight ratios and (**C**) lung weight/body weight ratios were determined after explantation of the organs following three weeks of angiotensin II (ATII) treatment. Accordingly, (**D**) animal weights, (**E**) heart weight/body weight ratios and (**F**) lung weight/body weight ratios were assessed after one week of isoprenaline (Iso) treatment.

The weights of the mice were affected neither by genotype nor by angiotensin II treatment (Figs. 4A, S4). The heart weight/body weight ratio was 38% higher in ATII-treated WT-Cre^+^ and Piezo2-KO mice than in untreated WT-Cre + and Piezo2-KO mice (i.e., without aggravation or attenuation by genotype) (Fig. 4B). Left ventricular weight development showed a similar picture (Fig. S5). In addition to the hearts, the lungs were considerably heavier after angiotensin II treatment, again similar in WT-Cre^+^ or Piezo2-KO mice (Fig. 4C). Isoprenaline treatment also had no effect on animal weight (Fig. 4D), but again, the heart weight/body weight ratio was higher in both genotypes but to a lesser extent than (the longer) angiotensin II treatment (Fig. 4E). Lung weights were not changed in the isoprenaline groups (Fig. 4F).

**Figure 5:**
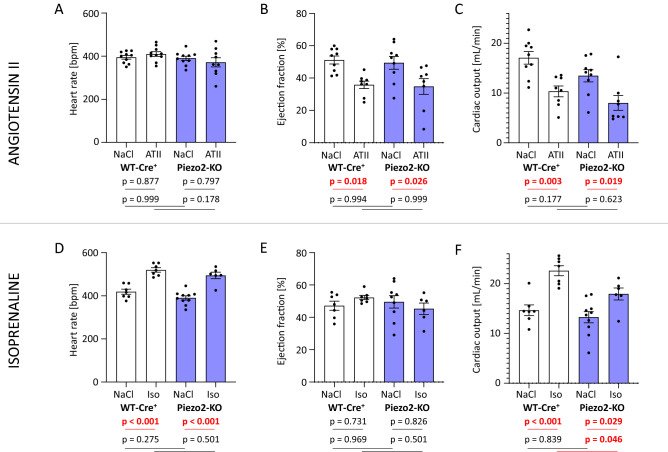
Heart function of WT-Cre^+^ and Piezo2-KO mice after angiotensin II or isoprenaline treatment. (**A**) Heart rate was assessed by electrocardiography, (**B**) ejection fraction and (**C**) cardiac output by echocardiography, all after three weeks of angiotensin II (ATII) treatment. Accordingly, (**D**) heart rate, (**E**) ejection fraction and (**F**) cardiac output were assessed after one week of isoprenaline (Iso)-treatment.

In terms of function, the heart rate of angiotensin II-treated mice was not changed (Figs. 5A, S6), while ejection fraction (Figs. 5B, S7) and cardiac output (Figs. 5C, S8) were markedly lower after angiotensin II. Here, again, WT-Cre^+^ and Piezo2-KO mice did not differ in their response to angiotensin II. Stroke volume and the directly measured parameter fractional shortening are depicted as additional parameters in Figs. S9 and S10. In contrast to angiotensin II, isoprenaline increased—as expected—the heart rates in WT-Cre^+^ and Piezo2-KO mice to a similar extent (Fig. 5D) but did not affect the ejection fraction (Fig. 5E). Accordingly, cardiac output was higher after isoprenaline treatment in both genotypes, whereas cardiac output after isoprenaline treatment in Piezo2-KO mice was lower than in WT-Cre^+^ mice (Fig. 5F).

**Figure 6:**
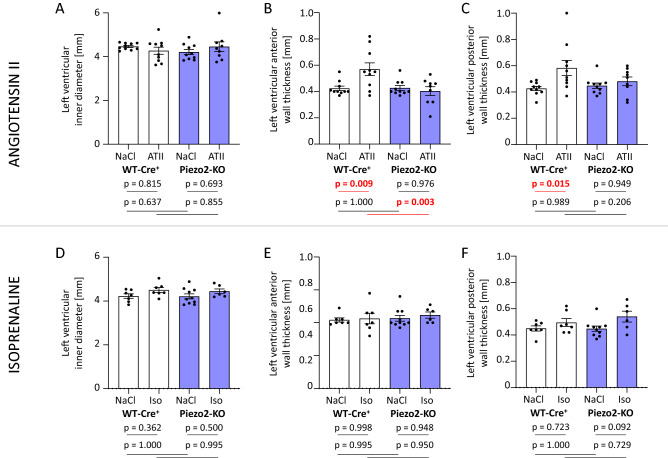
Dimensions of the left ventricle of WT-Cre^+^ and Piezo2-KO mice after angiotensin II or isoprenaline treatment. Echocardiographic assessment of (**A**) left ventricular inner diameter, (**B**) left ventricular anterior wall thickness and (**C**) left ventricular posterior wall thickness after three weeks of angiotensin II (ATII) treatment. Accordingly, (**D**) left ventricular inner diameter, (**E**) left ventricular anterior wall thickness and (**F**) left ventricular posterior wall thickness were assessed after one week of isoprenaline (Iso) treatment.

In addition to functional parameters, we analyzed the geometry of the left ventricles after treatment. Angiotensin II did not change the left ventricular inner diameter of WT-Cre^+^ or Piezo2-KO mice (Figs. 6A, S11), but it led to thickened anterior (Figs. 6B, S12) and posterior walls (Figs. 6C, S13) in WT-Cre^+^ mice. This effect was not observed in Piezo2-KO-mice. Isoprenaline treatment neither changed the inner diameter (Fig. 6D), nor anterior (Fig. 6E), nor posterior (Fig. 6F) wall thickness in any of the groups.

## Discussion

The piezo protein family drew our attention when we searched for a cardiomyocyte stress sensor that could complement the knowledge about load-induced short and long-term adaptations of the heart. Piezo1 and 2 are the only family members, and both have been described and named after their function (*piezein* = to press, Greek) in 2010^12^, while in earlier gene analyses, they appeared as family members 38A (piezo1) and 38B (piezo2). They have large molecular weights of approximately 300 kDa, are putatively glycosylated and form propeller-shaped homotrimers of almost 1 MDa. They are embedded in the cell membranes, not fully planar but rather with an inverted bell-like structure (opening up to the outside) in the side view^29,30^. This unusual structure leads to a very high tension sensitivity of the piezo channels, which act as Ca^2+^-permeable non-selective cationic channels^30^.

The first piezo report already mentioned the expression of both piezo proteins in a variety of tissues^12^. Piezo1 plays a prominent role in endothelial cells, which is supported by both the embryonic lethality of a global piezo1 knockout^31^ and by the lethality of a Tie2-driven endothelial-specific knockout in mice^32^. However, piezo1 is not restricted to endothelial cells, but it is also expressed in vascular smooth muscle cells^33^, fibroblasts, erythrocytes, adipocytes, T cells and other cell types^13^. Accordingly, the mechanosensing functions of piezo1 have been described in both non-cardiovascular (e.g., regulation of urinary osmolarity^34^, bladder urothelial filling sensor^35^, cancer cell migration^36^, bone formation^37^, erythrocyte integrity^38^) and cardiovascular functions (e.g., vascular development^31,32^, blood pressure regulation^39,40^). Piezo1 was proposed as a mechanosensor in cardiomyocytes in 2018^41^, but this early study has to be interpreted with caution, as the experiments were not performed in primary cardiomyocytes but in the proliferating cardiomyocyte-like fusion cell line AC16^41^. However, in a recent study^42^, the mechanosensing properties of piezo1 were also revealed in primary cardiomyocytes of murine hearts.

The high expression of piezo2 in dorsal root ganglia sensory neurons^12^ has fostered piezo2 research to be initially mainly centered on sensory neuron biology, gentle touch sensing (through its expression in the Merkel cell-neurite complex)^21,43^, itching^44^ and neuronal airway stretch sensing^45^. Consecutively, the expression and mechanotransducing effects of piezo2 have been revealed in endothelial cells promoting hyperalgesia^46^ and tumor angiogenesis^47^. However, latest with the exciting identification of piezo2 as the long-sought baroceptor (in conjunction with piezo1), piezo2 has been revealed as an important regulator in the cardiovascular system^48^.

Based on all these reports, both piezo proteins could either have the potential or have already been accounted^42^ to act as cardiomyocyte mechanosensors. Three aspects influenced our choice to assay piezo2 as mechanosensor: no (at least published) data on this question, detectable expression both in cardiac tissues and in cardiomyocytes, and piezo2 expression changes upon long-term cardiac stress. The latter could suggest an involvement in mechanosensing, although—of course—mechanosensors can also exert biological functions without expression changes. In retrospective and new analyses of control and mechanically challenged in vitro^16,17^ and in vivo rat tissue, we could only detect piezo2, but not 1, and furthermore, piezo 2 was clearly upregulated upon chronic stress, which could be confirmed in mouse cardiac tissue. In single-cell sequencing analyses of mouse^26^ and human^27^ hearts, both piezo transcripts were detectable in cardiomyocytes, while in a more detailed and newer human analysis, piezo2 was fourfold more highly expressed than piezo1 in ventricular cardiomyocytes^27^. Finally, “The Human Protein Atlas”^28^ reports moderate piezo2 cardiac protein abundance in a clear cardiomyocyte pattern, similar to what we observed in EHTs and hearts, but no cardiac piezo1.

Similar to piezo1, piezo2 constitutive knockout mice are not viable. Interestingly, they do not die in utero but perinatally^49^, which led us to speculate that the drastic hemodynamic changes after birth might be causative. To investigate the piezo2 effect in cardiomyocytes, we created a cardiomyocyte-specific piezo2 knockout by crossing α-MHC-Cre mice with floxed piezo2 mice. We aimed to address the question of whether piezo 2 in cardiomyocytes is meaningful in situations with short- or long-term cardiac mechanical stress, capitalizing on our repertoire of established stress models. Mechanical stress is linked to pathological conditions such as chronic arterial hypertension, which—untreated—is an important risk factor for the development of heart failure. Cationic channels have always been attractive targets for cardiovascular drugs (e.g., Na^+^, Ca^+^, K^+^-channels for antiarrhythmic drugs). Thus, the discovery of the piezo1-agonist Yoda 1^50^ and a chemical antagonist of Yoda 1 called Dooku 1^51^ is exciting, as it indicates druggability of the piezo channels in general. Specific piezo2-agonists or antagonists are still missing, but given the short time period needed to develop the piezo1 counterparts, there is good reason for optimism. Until then, only non-specific inhibitors of mechanosensitive channels, such as the spider toxin GsMTx4, have been employed^52^.

If cardiomyocyte-specific piezo2 knockout had an effect on fundamental phenomena such as Frank-Starling or Anrep, we would have anticipated serious implications at baseline, at least in homozygous mice. However, neither in heterozygous nor in homozygous mice, neither at birth nor later in life, we observed any differences from wild-type animals, including echocardiographically assessed heart function or dimensions. Thus, we proceeded and stressed the mice pharmacologically for longer periods of time. We chose the nonselective β-agonist isoprenaline to mimic increased sympathetic drive with hypercontractility and angiotensin II to mimic arterial vasoconstriction. Beneficial effects of a knockout under these pathological conditions only would have been attractive when thinking ahead of translating the findings into a future pharmacotherapy. As expected, isoprenaline slightly increased the heart weight/body weight ratio and, more clearly, the heart rate and cardiac output in WT-Cre^+^ mice. In piezo2 knockout mice, the effects of isoprenaline were almost identical to those in WT-Cre^+^ mice, with the exception that the increase in cardiac output was slightly less pronounced. Three weeks of angiotensin II treatment was intended to mimic clinical situations such as aortic constriction or arterial hypertension. Indeed, angiotensin II treatment of WT-Cre^+^ mice increased heart and lung weights (normalized to body weights) and cardiac wall thicknesses considerably, while ejection fraction and cardiac output decreased strongly, all indicating a decompensated, heart failure-like situation. Piezo2 knockout mice were not protected from detrimental angiotensin II effects and displayed similar functional and structural consequences. One notable exception was that the anterior and posterior left ventricular walls were not thicker in the angiotensin II-treated piezo2 knockout mice. The only differences of the piezo2 knock-out compared to WT-Cre^+^-mice (slightly lower cardiac output after isoprenaline-treatment, lower wall thicknesses after angiotensin II-treatment) were discrete and there was no clear evidence for a stronger cardiac dysfunction (e.g., increased lung weights) of the piezo2 knock-out-mice.

Taken together, the present data cannot exclude a minor contribution of piezo2 to the maintenance of cardiac function under conditions of increased cardiac stress but do not support our initial hypothesis that piezo proteins are major mechanosensors in the heart. Limitations have to be considered. (1) Methodological: Although published single-cell sequencing data displayed relatively low averaged concentrations of piezo2 mRNA in cardiomyocytes as well as in other cardiac cell types, it could be readily detected in all of our cardiac samples. The strong piezo2 immunostaining in our study and the Human Protein Atlas^28^ could argue for a relatively high protein stability. Interestingly, the single nuclei (sn)-sequencing data from the GTex Project suggests that a small fraction (≈ 5%) of cardiomyocytes expresses considerable amounts of piezo2 (above the still limited technical threshold of sn-sequencing). This corresponds to the unusual but yet unexplained patchy piezo2-staining pattern. Very low mRNA concentrations but remarkable protein abundance have also been reported for piezo1^42^. As the α-MHC promoter is active shortly after birth, recombination in our mice should start at this time point, and by the time of the long-term experiments (10–12 weeks after birth), piezo2 mRNA and protein will be almost completely absent. We verified the correct function of the Cre/loxP system by Sanger sequencing at critical points and the absence of piezo2 mRNA and protein in our mice by PCR or immunofluorescence staining. Further possible methodological problems typically occur with delivery of compounds by minipump and with regard to echocardiography. However, as the isoprenaline or angiotensin II-mice reproduced the expected structural and echocardiographic data, this appears unlikely. As these effects produced by these substances were all statistically significant, our study does not seem to be simply underpowered. (2) Wrong piezo chosen: We have expounded our reasons for the choice of piezo2 above. Nevertheless, the choice could have been wrong. At least more data currently suggest a role in cardiovascular regulation for piezo1 than for piezo2^13,42^. Further studies, especially long-term functional studies, could investigate the role of piezo1, potentially more simply taking advantage of the abovementioned piezo1 selective agonists and antagonists. (3) Piezo2-knockout alone is not sufficient: The piezo1/2 baroceptor study^48^ revealed that single knockouts had no effect, but that both piezo1 and 2 had to be knocked out to blunt baroreception. One might speculate that structurally similar piezo proteins serve as backups for each other^13^. (4) Piezo2 may act primarily in non-cardiomyocytes: We observed a clear upregulation of piezo2 in all stressed cardiac tissues that we analyzed. Compared to cardiomyocytes, piezo2 mRNA abundance appears to be slightly higher in cardiac endothelial cells and slightly lower in cardiac fibroblasts and smooth muscle cells^27^. Together with the assumption that endothelial cells outnumber cardiomyocytes by a factor of 3^53^, most piezo2-mRNA detected in cardiac tissues as well as its upregulation might stem from or occur in endothelial cells. As outlined above, we also observed piezo2 in this cell type, and furthermore, mechano-transducing effects of piezo2 in endothelial cells have been published.

In summary, a cardiomyocyte-specific piezo2 knockout in mice affects neither short-term cardiac function nor the long-term response to chronic angiotensin II- or isoprenaline stimulation.

## Supplementary Information

Supplementary Information.
