# Supplementary material for: Piezo2 is not an indispensable mechanosensor in murine cardiomyocytes
Source: Sci Rep. 2022 May 17;12:8193. doi: 10.1038/s41598-022-12085-9 (PMC9114012; doi:10.1038/s41598-022-12085-9)
Supplement: Supplementary file 1 — Supplementary Information. [file 41598_2022_12085_MOESM1_ESM.docx]

**- Supplemental Material -**

**Piezo2 is not an indispensable mechanosensor in murine cardiomyocytes**

Benjamin Kloth^1,2^, Giulia Mearini^1,3^, Florian Weinberger^1,3^, Justus Stenzig^1,3,^ Birgit Geertz^1,3^, Jutta Starbatty^1,3^, Diana Lindner^3,4^, Udo Schumacher^5^, Hermann Reichenspurner^2,3^, Thomas Eschenhagen^1,3^, Marc N. Hirt^1,3^

^1^Institute of Experimental Pharmacology and Toxicology, University Medical Center Hamburg-Eppendorf, Hamburg, Germany; ^2^Department of Cardiac Surgery, University Heart & Vascular Center, University Medical Center Hamburg-Eppendorf, Hamburg, Germany, ^3^DZHK (German Centre for Cardiovascular Research), partner site Hamburg/Kiel/Lübeck, Germany; ^4^Department of Cardiology, University Heart & Vascular Center, University Medical Center Hamburg-Eppendorf, Hamburg, Germany, ^5^Institute of Anatomy and Experimental Morphology, University Medical Center Hamburg-Eppendorf, Hamburg, Germany

Corresponding author:

Marc N. Hirt, Institute of Experimental Pharmacology and Toxicology, University Medical Center Hamburg-Eppendorf, Martinistraße 52, 20246 Hamburg, Germany, Tel: +49-40-7410-52180, Fax: +49-40-7410-54876; E-mail: m.hirt@uke.de

# Additional Methods

## Isolation of RNA and quantitative PCR

Mouse or rat cardiac tissue samples as well as rat EHT were homogenized with steel beads in a highly chaotropic isolation buffer. A phenol-chloroform based method (TRIzol, Ambion) was performed and total RNA was extracted from the aqueous phase utilizing isopropanol and silica columns (Lexogen Split RNA Extraction Kit). Determination of the nucleic acid concentration was performed by measuring the absorbance at 260 nm using a Nanodrop 2000c spectrophotometer. RNA was stored at -80 °C for further analyses. For reverse transcription of mRNA the High Capacity cDNA Reverse Transcription kit (Applied Biosystems) was used according to the manufacturer’s instructions. 250 ng total RNA isolated from engineered heart tissue or 1 µg total RNA isolated from cardiac tissue were reversely transcribed for 2 h at 37 °C followed by an inactivation step for 5 min at 85 °C. The resulting cDNA was diluted to a final working concentration of 1.25 ng/µL for engineered heart tissue and 10 ng/µL for cardiac tissue samples.

The semi-quantitative PCRs (Figs. 1A and 3C) were performed with 35 cycles and the HOT FIREPol Ready to Load reagents (Solis Biodyne) according to the manufacturer’s instructions. PCR products were visualized on a 2%-agarose gel with Midori Green Advance DNA stain (NIPPON Genetics).

The primer sequences for rat cardiac tissue were:

Piezo1 forward: 5’-GCCCCTCAAGACACAGCATA-3’

Piezo1 reverse: 5’-CACGTTTGCCCAAAGGTTACA-3’

Piezo2 forward: 5’-ATAAAGAAGGGCAACGTTCCGA-3’

Piezo2 reverse: 5’-AGCAGGGTTGCTTCAGTGTAA-3’

GAPDH forward: 5’-CTCATGACCACAGTCCATGC-3’

GAPDH reverse: 5’-TTCAGCTCTGGGATGACCTT-3’

The primer sequences for mouse cardiac tissue were:

Piezo2 (exon 43-45)

forward: 5’-TTTGGGAAACACTCGGCAG-3’

Piezo2 (exon 43-45)

reverse: 5’-TGAATCCCAAACACGAGGATGA-3’

GAPDH forward: 5’-ATTCAACGGCACAGTCAAG -3’

GAPDH reverse: 5’-TGGCTCCACCCTTCAAGT-3’

The quantitative PCRs (Fig. 1 B+C) were performed with an ABI Prism 7900 HT Sequence Detection System (Applied Biosystems). To determine RNA expression levels of various genes, real-time PCR was carried out using 5 µL of gene expression master mix (Thermo Fisher Scientific) and 0.5 µL of the gene expression assay for Piezo2 (Mm01265861_m1) purchased from Thermo Fisher Scientific. The assays for gene expression consists of gene specific forward and reverse primers and the FAM-labelled probe. As template 1 µL cDNA was used in a final volume of 10 µL, each performed in duplicates. As an endogenous control, the gene expression of 18S (Hs99999901_s1) and Cdkn1b (Mm00438167_g1) was assessed. The gene expression data were first normalized to the housekeeping genes using the ΔCt-method and second to the mean of the control animals at 2 weeks (Fig. 1B) or at 3 weeks (Fig. 1C) using the ΔΔCt-method. Finally, the mean of control animals at 2 weeks (Fig. 1B) or at 3 weeks (Fig. 1C) were set to 1.

## Genotyping of mice for floxed piezo2-locus and/or α-MHC-Cre

All mice in this project were genotyped. DNA was extracted from mouse tail tips with the REDExtract-N-Amp Tissue PCR Kit Protocol (Sigma-Aldrich XNAT) according to the manufacturer’s instructions. The PCR was performed at 59 °C annealing temperature for 30 cycles.

A mix of the following 3 primers was used to identify piezo2 wild-type or floxed alleles:

P2 forward: 5’-AGGCTCAGACTTGGAGATCCTGTAGCA-3’

P2 wild type reverse: 5’-CCCTACCCACCCATTCCCATTTTT-3’

P2 floxed reverse:   5’-GACTCAGATTTTCCACATGGGGGTACTA-3’

A 131 bp PCR-product can be yielded from a piezo2 WT allele, and a 258 bp PCR-product from a floxed piezo2 allele.[^1^](#_ENREF_1)

The following primers were used to identify α-MHC-Cre^+^-mice:

Cre forward: 5’- GTTCGCAAGAACCTGATGGAC-3’

Cre reverse: 5’- CTAGAGCCTGTTTTGCACGTT-3’

A 340 bp PCR-product can be yielded from an α-MHC-Cre positive allele.

## Long range PCR

Genomic DNA was extracted from mouse ventricles with the REDExtract-N-Amp Tissue PCR Kit Protocol (Sigma-Aldrich XNAT) according to the manufacturer’s instructions. The DNA amplification was also performed with this kit with the exception that the kit’s polymerase was replaced by the PrimeStar HS DNA Polymerase (Takara, R010B).

The primer sequences were as follows: Piezo2 long range PCR forward primer (5’-TAGCACTTAGATGGGGCAGG-3’; 4,488-4,507), Piezo2 long range PCR reverse primer (5’-AGAGGGTGGGCATCTTTTGT-3’; 7,655-7,674). The loxP-site positions were 4,623-4,656 and 7,089-7,122, the floxed exons of piezo2 were: exon 43 (5,011-5,143), exon 44 (6,215-6,362), exon 45 (6,467-6,681).

## Isolation and purification of cardiomyocytes from adult murine hearts by Langendorff perfusion and albumin gradient sedimentation

Isolation of cardiomyocytes in a Langendorff perfusion setup was performed according to a previously published protocol.[^2^](#_ENREF_2) Briefly, murine hearts were mounted on a temperature-controlled Langendorff system, perfused with a Ca^2+^-free buffer (113 mM NaCl, 4.7 mM KCl, 0.6 mM KH_2_PO_4_, 0.6 mM Na_2_HPO_4_, 1.2 mM MgSO_4_, 12 mM NaHCO_3_, 10 mM KHCO_3_, 30 mM taurine, 5.5 mM glucose, 10 mM 2,3-butanedione monoxime (BDM), 10 mM HEPES, pH 7.46) at 37 °C, and then digested for 10 min in the Ca^2+^-free solution containing 0.1 mg/mL Liberase Blendzyme (Roche Diagnostics). The ventricles were then minced to dissociate single cells.

For albumin gradient centrifugation 6% albumin containing perfusion buffer was prepared. 5 mL of cardiomyocyte containing solution was pipetted on top of 5 mL albumin solution. After a sedimentation time of 15 minutes the supernatant was carefully removed. The cell pellet was washed with perfusion buffer and a second round of sedimentation followed. Finally, Ca^2+^ was slowly readjusted.

# Additional Figures

**Fig. S1** Low and medium magnification images of immunohistochemical piezo2-stainings and isotype control of rat cardiac tissue.

**Fig. S2** Immunofluorescent co-stainings of piezo2 (green), wheat germ agglutinin (WGA, membranes, red) and DAPI (nuclei blue) in **A** longitudinal sections of murine left ventricles additionally stained for troponin I (orange), and in **B** cross-sections with a focus on vascular structures additionally stained for α-smooth muscle actin (orange). The white rectangle in A displays a zoomed-in region.

**Fig. S3** Basal characteristics of WT (n=4), Het Piezo2-KO (n=2) and Piezo2-KO (n=4) mice. Animal weight (A), heart rate (B), ejection fraction (C), left ventricular inner diameter (D), left ventricular anterior (E) and posterior (E) wall thickness were analyzed under non-stressed conditions. B was assessed by electrocardiogram, C-F by echocardiography.

**~~~~ Fig. S4** Animal weights of WT-Cre^+^ and Piezo2-KO mice before (A) and after three weeks of continuous angiotensin II (ATII)-administration (B) and before (C) or after one week of isoprenaline (Iso)-administration (D). Both drugs were administered via osmotic minipumps and NaCl-loaded minipumps served as controls. Each dot represents a single mouse, groups consisted of 6-10 mice.

**Fig. S5** Left ventricular weights of WT-Cre^+^ and Piezo2-KO mice assessed after three weeks of continuous angiotensin II (ATII)-administration (A) or after one week of isoprenaline (Iso)-administration (B). Both drugs were administered via osmotic minipumps and NaCl-loaded minipumps served as controls. Each dot represents a single mouse, groups consisted of 6-10 mice.

**~~~~ Fig. S6** Heart rate of WT-Cre^+^ and Piezo2-KO mice assessed by electrocardiogram before (A) and after three weeks of continuous angiotensin II (ATII)-administration (B) and before (C) or after one week of isoprenaline (Iso)-administration (D). Both drugs were administered via osmotic minipumps and NaCl-loaded minipumps served as controls. Each dot represents a single mouse, groups consisted of 6-10 mice.

**~~~~ Fig. S7** Echocardiographic assessment of ejection fraction of WT-Cre^+^ and Piezo2-KO mice before (A) and after three weeks of continuous angiotensin II (ATII)-administration (B) and before (C) or after one week of isoprenaline (Iso)-administration (D). Both drugs were administered via osmotic minipumps and NaCl-loaded minipumps served as controls. Each dot represents a single mouse, groups consisted of 6-10 mice.

**~~~~ Fig. S8** Echocardiographic assessment of cardiac output of WT-Cre^+^ and Piezo2-KO mice before (A) and after three weeks of continuous angiotensin II (ATII)-administration (B) and before (C) or after one week of isoprenaline (Iso)-administration (D). Both drugs were administered via osmotic minipumps and NaCl-loaded minipumps served as controls. Each dot represents a single mouse, groups consisted of 6-10 mice.

**~~~~ Fig. S9** Echocardiographic assessment of stroke volume of WT-Cre^+^ and Piezo2-KO mice before (A) and after three weeks of continuous angiotensin II (ATII)-administration (B) and before (C) or after one week of isoprenaline (Iso)-administration (D). Both drugs were administered via osmotic minipumps and NaCl-loaded minipumps served as controls. Each dot represents a single mouse, groups consisted of 6-10 mice.

**~~~~ Fig. S10** Echocardiographic assessment of fractional shortening of WT-Cre^+^ and Piezo2-KO mice before (A) and after three weeks of continuous angiotensin II (ATII)-administration (B) and before (C) or after one week of isoprenaline (Iso)-administration (D). Both drugs were administered via osmotic minipumps and NaCl-loaded minipumps served as controls. Each dot represents a single mouse, groups consisted of 6-10 mice.

**~~~~ Fig. S11** Echocardiographic assessment of left ventricular inner diameter of WT-Cre^+^ and Piezo2-KO mice before (A) and after three weeks of continuous angiotensin II (ATII)-administration (B) and before (C) or after one week of isoprenaline (Iso)-administration (D). Both drugs were administered via osmotic minipumps and NaCl-loaded minipumps served as controls. Each dot represents a single mouse, groups consisted of 6-10 mice.

**~~~~ Fig. S12** Echocardiographic assessment of left ventricular anterior wall thickness of WT-Cre^+^ and Piezo2-KO mice before (A) and after three weeks of continuous angiotensin II (ATII)-administration (B) and before (C) or after one week of isoprenaline (Iso)-administration (D). Both drugs were administered via osmotic minipumps and NaCl-loaded minipumps served as controls. Each dot represents a single mouse, groups consisted of 6-10 mice.

**~~~~ Fig. S13** Echocardiographic assessment of left ventricular posterior wall thickness of WT-Cre^+^ and Piezo2-KO mice before (A) and after three weeks of continuous angiotensin II (ATII)-administration (B) and before (C) or after one week of isoprenaline (Iso)-administration (D). Both drugs were administered via osmotic minipumps and NaCl-loaded minipumps served as controls. Each dot represents a single mouse, groups consisted of 6-10 mice.

**Fig. S14** Uncropped images of agarose gels depicted in the main Figs 1A, 3B, 3C. The lanes in the uncropped image of Fig. 3B, which are not included in main Fig. 3B, are additional homozygous and heterozygous Piezo2-KO animals.

# References

1. Woo SH, Ranade S, Weyer AD, Dubin AE, Baba Y, Qiu Z, Petrus M, Miyamoto T, Reddy K, Lumpkin EA, Stucky CL and Patapoutian A. Piezo2 is required for Merkel-cell mechanotransduction. *Nature*. 2014;509:622-6.

2. Pohlmann L, Kroger I, Vignier N, Schlossarek S, Kramer E, Coirault C, Sultan KR, El-Armouche A, Winegrad S, Eschenhagen T and Carrier L. Cardiac myosin-binding protein C is required for complete relaxation in intact myocytes. *Circ Res*. 2007;101:928-38.
